# Street Collections

**Published:** 1897-07-10

**Authors:** 


### Editor's Letter-Box.

[Our Correspondents are reminded that prolixity is a great bar to publication, and that brevity of style and conciseness of statement greatly facilitate early insertion.]

### STREET COLLECTIONS.

Mr. W. G. Towler writes: Notwithstanding the wholesale condemnation of the system of street collections on behalf of various charities, the practice still obtains throughout the metropolis. To-day some thousands of persons are importuning passers-by at every street corner to contribute to the Hospital Saturday Fund. It has been suggested that the County Council should pass bye-laws regulating these collections, but I believe a more excellent way of avoiding the evils of the present method would be to provide each collector with specially designed buttons like the election buttons used in the United States during the last Presidential election. Each contributor would, on giving his mite, receive from the hands of the box-holder one of these buttons, which he or she would wear in a conspicuous position. Other collectors would be careful to note this badge of virtue and avoid troubling the wearer. Thus the great annoyance of being accosted some fifty times in the course of a journey across London would disappear, a great deal of energy which is now wasted by collectors would be conserved, and the most important objection to street collections would cease to exist. People who subscribe in other ways could also be protected by this means; only the non-subscriber need be harassed. Let him object if he likes; he should be without the pale of the law.

